# Pinpointing regulatory protein phosphatase 2A subunits involved in beneficial symbiosis between plants and microbes

**DOI:** 10.1186/s12870-021-02960-4

**Published:** 2021-04-16

**Authors:** Irina O. Averkina, Muhammad Harris, Edward Ohene Asare, Berenice Hourdin, Ivan A. Paponov, Cathrine Lillo

**Affiliations:** 1 IKBM, Department of Chemistry, Bioscience and Environmental Engineering, University of Stavanger, 4036 Stavanger, Norway; 2 Faculty of Veterinary Medicine, Norwegian University of Life Sciences, 1433 Ås, Norway; 3 NIBIO, Norwegian institute of Bioeconomy Research, Division of Food Production and Society, P.O. Box 115, NO-1431 Ås, Norway; 4 Current address: Department of Food Science, 8200 Aarhus University, Aarhus, Denmark

**Keywords:** Abscisic acid, *Azospirillum brasilense*, *Funneliformis mosseae*, Gibberellin, Mycorrhiza, PP2A, PGPR, *Pseudomonas simiae*, *Rhizophagus irregularis*, Tomato

## Abstract

**Background:**

PROTEIN PHOSPHATASE 2A (PP2A) expression is crucial for the symbiotic association between plants and various microbes, and knowledge on these symbiotic processes is important for sustainable agriculture. Here we tested the hypothesis that PP2A regulatory subunits, especially *B’φ* and *B’θ,* are involved in signalling between plants and mycorrhizal fungi or plant-growth promoting bacteria.

**Results:**

Treatment of tomato plants (*Solanum lycopersicum)* with the plant growth-promoting rhizobacteria (PGPR) *Azospirillum brasilense* and *Pseudomonas simiae* indicated a role for the PP2A B’θ subunit in responses to PGPR. Arbuscular mycorrhizal fungi influenced *B’θ* transcript levels in soil-grown plants with canonical arbuscular mycorrhizae. In plant roots, transcripts of *B’φ* were scarce under all conditions tested and at a lower level than all other PP2A subunit transcripts. In transformed tomato plants with 10-fold enhanced *B’φ* expression, mycorrhization frequency was decreased in vermiculite-grown plants. Furthermore, the high *B’φ* expression was related to abscisic acid and gibberellic acid responses known to be involved in plant growth and mycorrhization. *B’φ* overexpressor plants showed less vigorous growth, and although fruits were normal size, the number of seeds per fruit was reduced by 60% compared to the original cultivar.

**Conclusions:**

Expression of the *B’θ* gene in tomato roots is strongly influenced by beneficial microbes. Analysis of *B’φ* overexpressor tomato plants and established tomato cultivars substantiated a function of *B’φ* in growth and development in addition to a role in mycorrhization.

**Supplementary Information:**

The online version contains supplementary material available at 10.1186/s12870-021-02960-4.

## Background

Plants are colonized by a wide range of microorganisms, beneficial as well as harmful [1–3]. The beneficial microorganisms such as plant growth-promoting rhizobacteria (PGPR) and arbuscular mycorrhizal fungi (AMF) can improve nutrient acquisition and water uptake and protect the host against pathogens and abiotic stress. The effects of PGPR have been studied for many years and frequently used bacterial model species are *Azospirillum brasilense* and *Pseudomonas simiae* [4–6]. *A. brasilense* already has practical applications in agriculture [5], and field studies have shown that certain pseudomonads strains and arbuscular mycorrhizal fungi can increase yield and quality of tomato plants grown under sub-optimal fertilization conditions [7]. Mycorrhizae are a symbiosis between plants and fungi that evolved early, about 460 million years ago, and is likely to have been important for plants to colonize land. Mycorrhizae are important for uptake of nutrients, tolerance to drought, and may also be important for improving defence against pathogens. Arbuscular mycorrhizae (AM) are the most common type of mycorrhizae and more than 85% of land plant species can form arbuscular mycorrhizae. The mechanisms of signalling between plants and microbes, establishment and upholding of symbiosis are still far from understood.

Protein phosphatase 2A is a major protein phosphatase in plants and is involved in regulation of metabolism, development, stress responses and interactions with microbes [8–10]. The PP2A complex is made up of three canonical subunits, a catalytic (C), a scaffolding (A) and a regulatory (B) subunit. In tomato there are at least five putative C, three A, and 15 B subunits [11]. The large number of B subunits is important for the various PP2A complexes to be specific for different substrates and cellular localizations [12, 13]. PP2A involvement in microbe and plant symbiosis is evident from work with the maize pathogenic bacterium *Pantoea stewartii* and the broad host (including tomato) pathogen *Phytophtora capsi* since both pathogens produce effectors that weaken the defence of plants by interacting with PP2A subunits [14, 15]. In yeast-two-hybrid screening, the effector from *P. stewartii* interacted with a maize regulatory B′ subunit, and the effector from *P. capsi* interacted with scaffolding A subunits from pepper, *Nicotiana* spp. and *Arabidopsis*. The PP2A catalytic subunits divide into two clades in higher plants, and the two genes *C1*and *C2* form one clade in tomato (Subfamily 1). The Subfamily 1 was previously found to be involved in responses to bacterial treatments in both tomato and potato plants. In tomato, *Pseudomonas syringae* enhanced expression of the catalytic subunit *C1*, and in *Nicotina benthamiana* silencing of the closely related catalytic subunits of Subfamily 1 showed that they were involved in defence responses [16]. In the present study, both Subfamily 1 genes of tomato were included in expression analysis.

When evaluating a range of plant species, the ability to make mycorrhizae was found to correlate with possession of a regulatory PP2A subunit called B’φ [11]. The B’φ clade is evolutionary very old and has not expanded, indicating that it is involved in some basic functions. The *B’φ* clade has not been much studied except for a couple of investigations using *Medicago sativa* and *M. truncatula* [17, 18], and to our knowledge no studies have been performed with tomato *B’φ.* Based on the work referred above, we selected the *B’φ* gene as a candidate for being involved in the formation of mycorrhizae in tomato. Hormones, including abscisic acid (ABA) and gibberellins (GA), are important for development of AM in tomato roots [19]. ABA also participates in the induction of AM associated with PP2A expression in *M. truncatula* [17]. Since ABA is important in AM formation, the tomato *Bβ (clade III)* gene was included as an orthologue of the ABA-induced gene in *Medicago* spp. [18]. Expression of reporter genes for ABA and GA responses were also assayed in the present study. In *Arabidopsis*, *B’θ* had previously been detected to be involved in the response to microbial treatment [20], and its closest orthologue in tomato was therefore included in all expression experiments here. To shed light on physiological function of PP2A in plant-microbe symbiosis, tomato plants were grown in vermiculite or soil and treated with PGPR or AMF. Transcripts of the selected PP2A subunits were tested in tomato (cv. Heinz) and also in the Heinz cultivar transformed to overexpress *B’φ* and used to study mycorrhizal colonization.

## Results

### Expression of *PP2A* subunit genes in different tissues

**Fig. 1:**
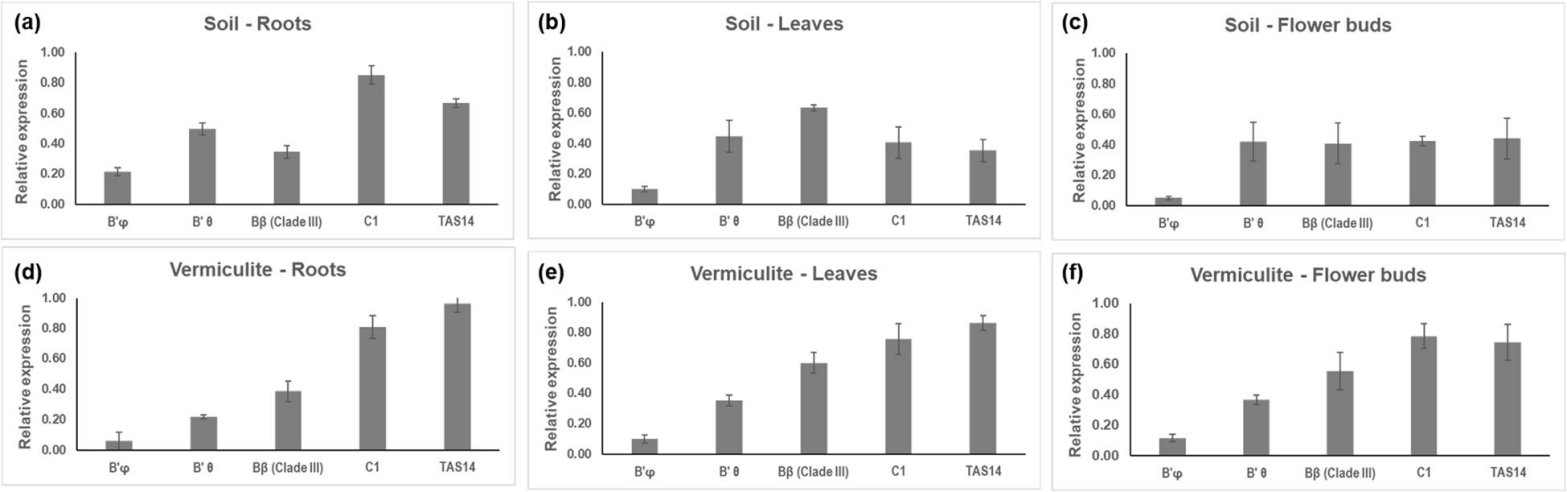
Gene expression of selected PP2A subunits in different tissues from 3.5-month-old-tomato plants grown in soil or vermiculite and determined by sqRT-PCR. **a, b, c** Plants in soil; and **d, e, f** Plants in vermiculite. Transcript levels of *B’φ, B’θ*, *Bβ (clade III)*, *C1* and *TAS14* were measured in (**a, d**) Roots; **b, e** Leaves; and **c, f** Flower buds. Values denote the average level of expression from three biological replicates normalized by the reference gene *ACTIN41*. Mean values ±SE are shown

Expression levels of selected *PP2A* subunit genes and *TAS14* as an ABA responsive gene [21] are presented in Fig. 1. Strikingly, *B’φ* was expressed at very low levels in all tissues tested, in some tissues barely detectable. Expression of *B’θ* was not much influenced by type of tissue but was always lower in vermiculite-grown plants compared with soil-grown plants, the lowest value was in roots of vermiculite-grown plants. The *Bβ (clade III)* gene showed its lowest expression in roots, highest in leaves and medium values in flower buds, but showed no correlation with the ABA reporter gene. The *C1* gene was highly expressed in roots in both soil and vermiculite, but more moderately in leaves and buds of soil-grown compared with vermiculite-grown plants. *TAS14* was always more highly expressed in vermiculite compared with soil, indicating more ABA or higher ABA sensitivity in vermiculite-grown plants. Publicly available expression analysis from the Sol genetics database [22] revealed similar expression patterns for these genes in roots and leaves in *S. lycopersicum* seedlings, confirming very low levels of *B’φ* in all tissues (Supplemental S1Fig. a, b).

### Effects of PGPR treatment

**Fig. 2:**
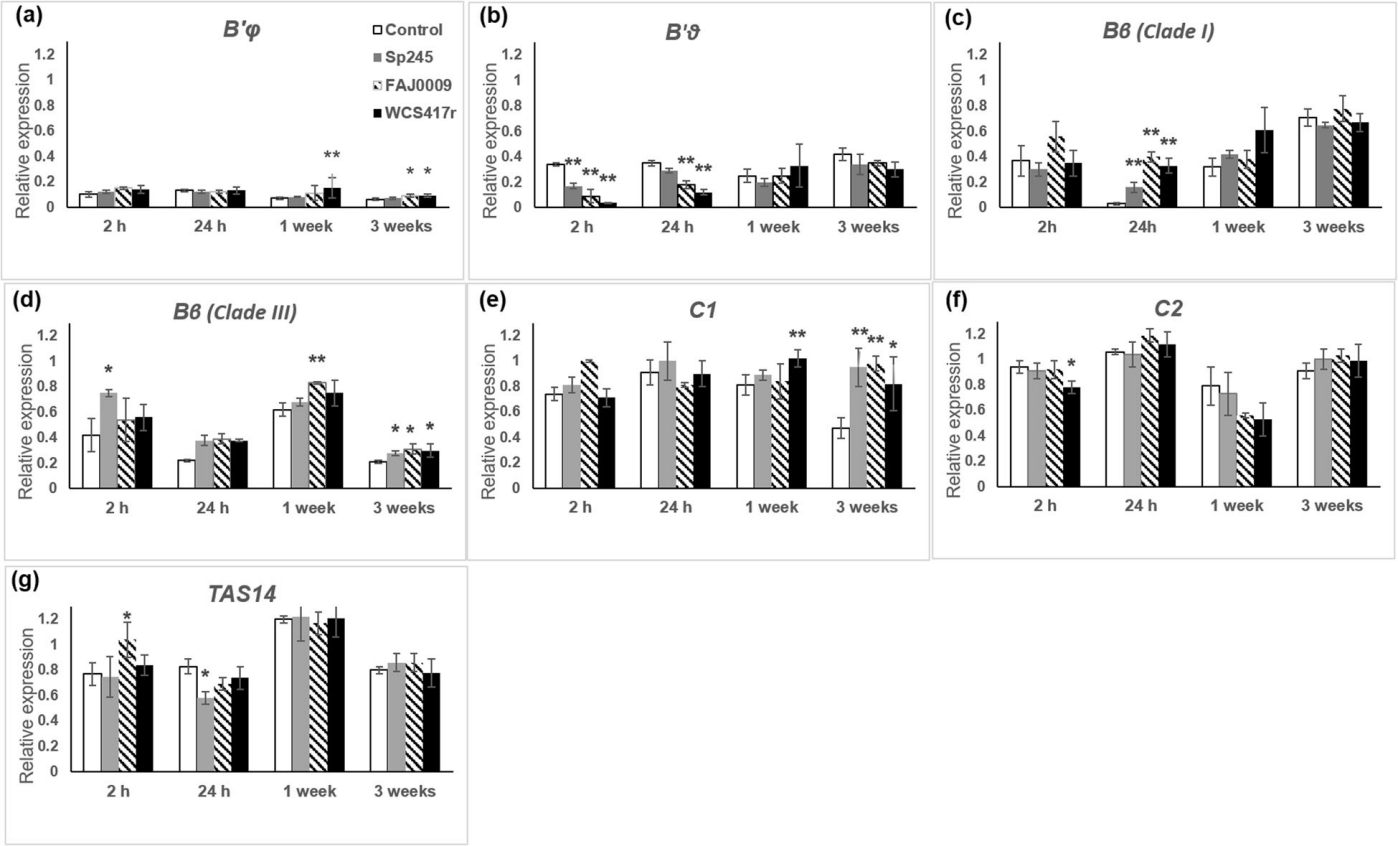
Time course for expression of selected *PP2A* subunits and *TAS14* in tomato roots inoculated with PGPR. Tomato plants were grown in vermiculite for 40 days. Thereafter, treated with 10 mM MgSO_4_ (control) (white bars), Sp245 (grey bars), FAJ0009 (hatched bars) and WCS417r (black bars) and harvested after 2 h, 24 h, 1 week and 3 weeks. Genes analysed were (**a**) *B’φ*; (**b**) *B’θ*; (**c**) *Bβ (clade I)*; (**d**) *Bβ (clade III)*; (**e**) *C1*; (**f**) *C2;* (**g**) *TAS14*. Columns marked with one or two asterisks are significantly different from the corresponding control according to student’s t-test at *p*-value < 0.1 or 0.05 respectively

Gene expression was studied in roots from plants grown in vermiculite and treated with three strains of PGPR, *A. brasilense* Sp245, *A. brasilense* FAJ0009 (auxin producing deficient mutant) and *P. simiae* WCS417r. Samples were harvested from 2 h to 3 weeks after inoculation (Fig. 2). *B’φ* expression was slightly increased one week after treatment with *P. simiae* WCS417r but was still the gene expressed at the lowest level compared with all other genes tested (Fig. 2a-g). The similar effects of *A. brasilense* and its auxin deficient mutant strain FAJ0009 on *B’φ* expression indicates that the expression of this gene is independent of auxin. A most striking result was the transiently decreased expression of *B’θ* after 2 h and 24 h in bacteria-treated roots, especially in response to *P. simiae* WCS417r (Fig. 2b). This response was also auxin independent because *A. brasilense* auxin-deficient FAJ0009 induced a similar or stronger effect than wild type *A. brasilense*. *B’θ* expression then regained control levels after 1 and 3 weeks. For the *Bβ (Clade I)* gene, expression was decreased in the control after 24 h, but this decrease was largely prevented by bacterial treatments. The control then regained activity, and after 3 weeks there was no difference between control and inoculated plants (Fig. 2c). For *Bβ (Clade III)* there was also a tendency that bacterial-treated plants showed higher expression than the control (Fig. 2d). Expression of *C1* decreased after 3 weeks in control plants, but this was prevented in the bacteria-treated plants (Fig. 2e). Expression of the *C2* gene and the ABA reporter gene were not much influenced by any of the bacteria strains (Fig. 2f, g).

**Fig. 3:**
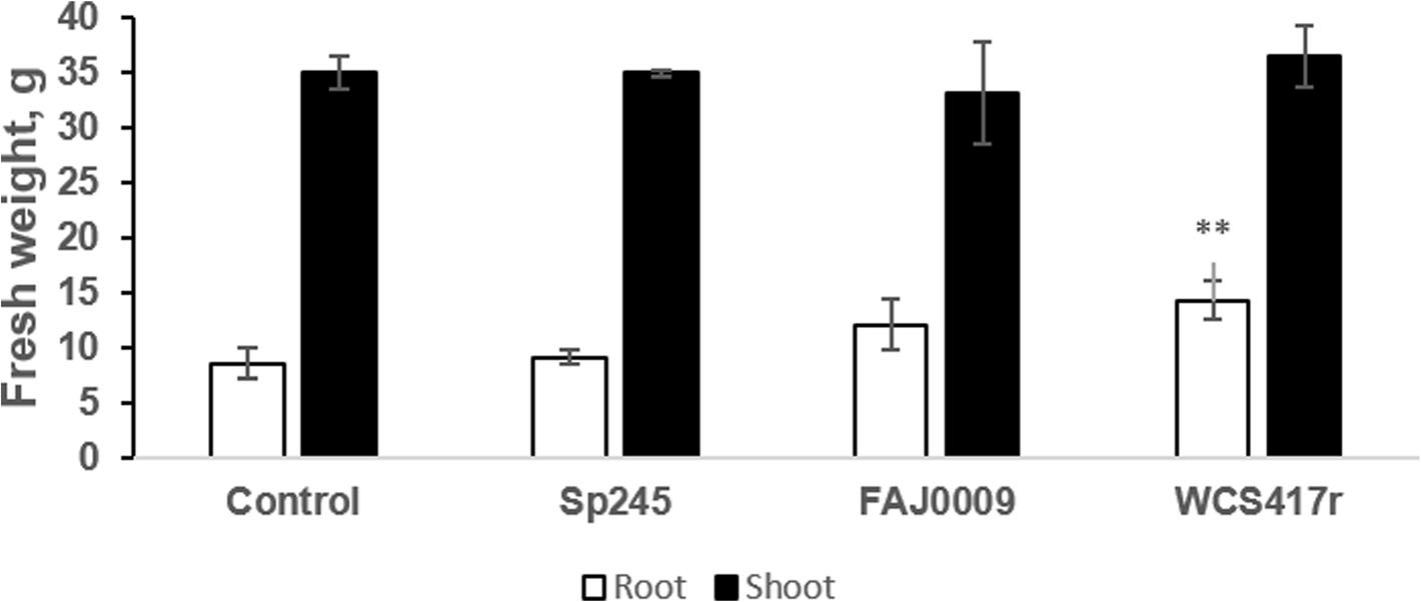
Fresh weight of plants after inoculation with PGPR and grown in vermiculite for three weeks. Tomato roots are represented by white bars and shoots by black bars. Data are means ± SE of 4 plants (*n* = 4). The bar marked with two asterisks is significantly different from the control plants according to student’s t-test at *p*-value < 0.05. Pictures of plants are in Fig. S2

Fresh weight of roots and shoots was measured three weeks after inoculation and showed that roots of PGPR-treated plants had higher fresh weight than control plants, but this was significant only for WCS417r (Fig. 3). This effect was independent of auxin, as inoculation with the auxin-deficient FAJ0009 strain showed rather stronger but not significant increase in root growth compared with the Sp245 strain.

### Effects of colonization by AMF

**Fig. 4:**
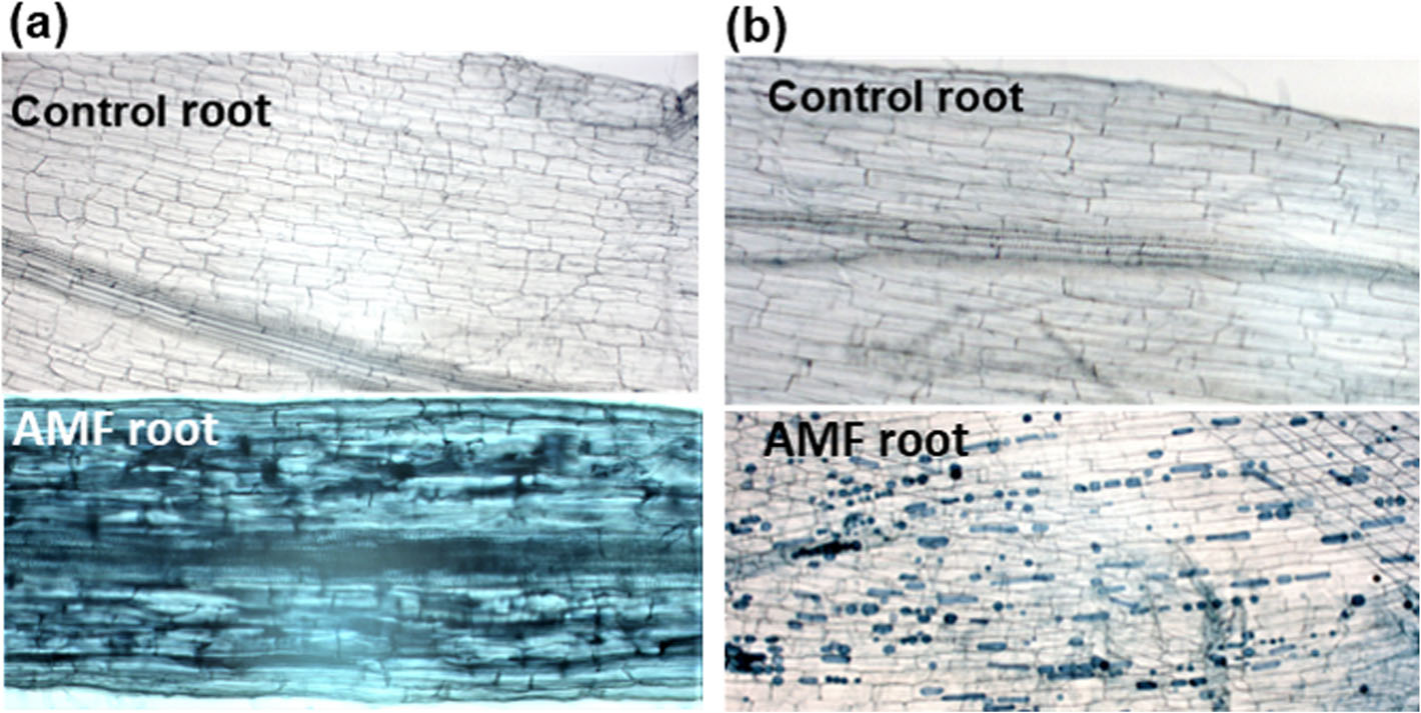
Morphology of AM colonization in tomato roots stained with trypan blue 3.5 months after inoculation with AMF. Bright-field images of roots grown in (**a**) soil and in (**b**) double autoclaved vermiculite

Tomato plants were inoculated with AMF in both soil and vermiculite. Since establishment of mycorrhizae is a time-requiring process, samples for gene expression were harvested 3.5 months after planting and inoculation with AMF. Microscopy analysis showed that the type of growing medium strongly influenced AM morphology. Roots grown in soil formed canonical AM usually observed when roots are colonized by more than one AMF species [23] (Fig. 4a). Roots in vermiculite formed vesicular mycorrhizae (VM) (Fig. 4b). No AM was observed in the control plants.

**Fig. 5:**
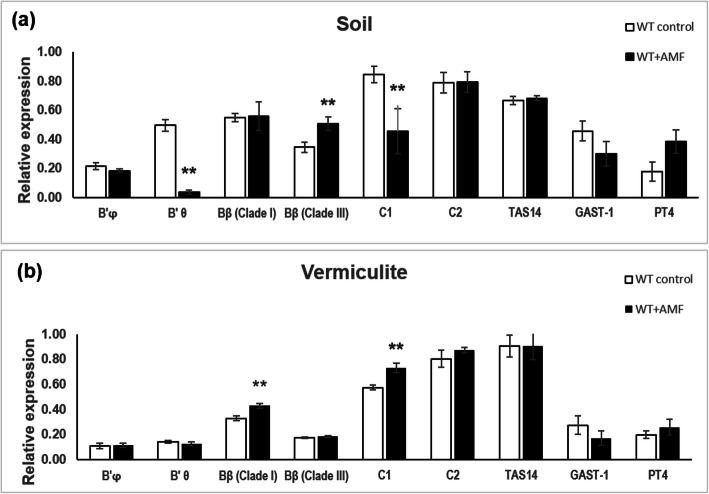
Expression of *PP2A* subunit genes and AM-associated genes in roots not treated or treated with AMF. Tomato plants had been grown for 3.5 months after the mock treatment (white bars) and AMF treatment (black bars) and were grown in (**a**) soil or (**b**) vermiculite. The values are averages from three biological replicates normalized by the reference gene *ACTIN41.* Values marked with two asterisks are significantly different from the corresponding control according to student’s t-test at *p*-value < 0.05, *n* = 3

The selected PP2A subunit genes were tested in plants grown in soil and vermiculite after treatment with AMF. A reporter gene for GA levels, *GAST1* (*GA-STIMULATED TRANSCRIPT 1*) was included in the analysis [19, 24]. *PT4* (*PHOSPHATE TRANSPORTER 4*) was included as a reporter gene for mycorrhizae-inducible inorganic phosphate transporter and is a marker for AM symbiosis [25]. The *PT4* gene was up-regulated after addition of AMF by 116% for soil-grown plants, in agreement with AM formation (Fig. 5a). The up-regulation in vermiculite-grown plants was only 30% and this may be explained by the formation of VM instead of AM. *TAS14* expression was higher in plants grown in vermiculite than in soil, as previously observed (Figs. 1, and 5). After AMF inoculation, *TAS14* remained constant, while the data indicated that *GAST* was down-regulated, suggesting that the ABA to GA ratio was changed in favour of mycorrhizae formation [24]. These experiments confirmed that *B’φ* was the *PP2A* gene expressed at the lowest level, with especially low values in vermiculite-grown plants (Figs. 1, and 5). The expression of *B’φ* was not influenced by AMF. The expression of *B’θ* in roots was significantly lower in control plants grown in vermiculite compared with soil (Fig. 5), in agreement with previous experiments (Fig. 1a, d). Strikingly, as for PGPR treatment, *B’θ* was down-regulated by AMF treatment, though only in soil-grown plants where control plants had high levels of *B’θ* transcripts (Fig. 5a). The *Bβ (Clade III)* was up-regulated in soil, whereas *Bα (Clade I)* was up-regulated in vermiculite in AMF-treated plants. *C1* also showed different responses to AMF in soil and vermiculite. *C2* was not influenced by AMF treatment.

### *B’φ* overexpressor plants

**Fig. 6:**
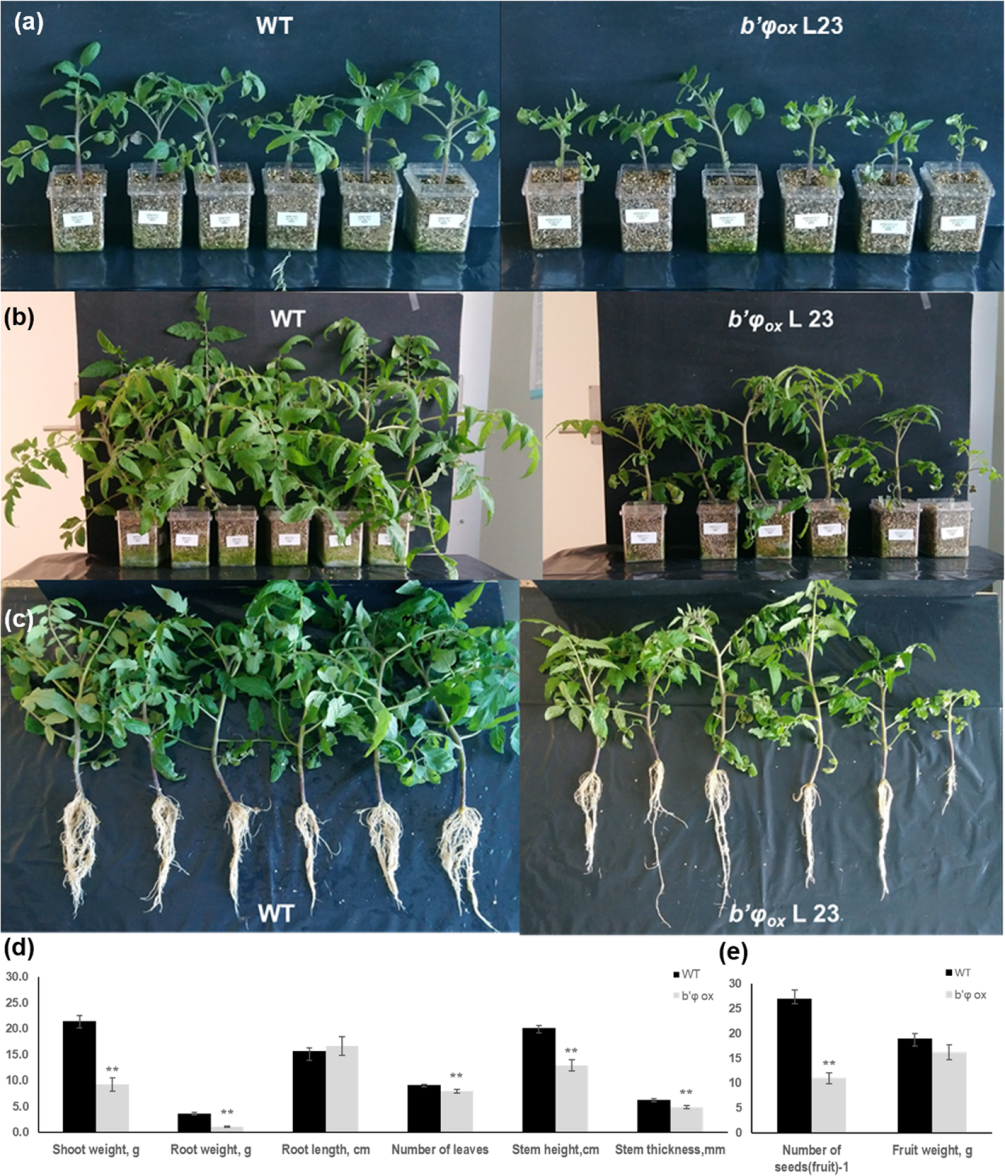
Phenotypic characterization of *b’φ*_*ox*_ **(**over-expressor). Original genotype **(**WT) and *b’φ*_*ox*_ plants. **a** 25 days old; **b, c** 8 weeks old; **d** Mean values of shoot weight, root weight, root length, number of leaves and stem height for 12 WT plants (black columns) and 18 *b’φ*_*ox*_ plants (grey columns) (F_1_ of three mutant lines); **e** Fruit fresh weight and seed number are from tomato fruits collected after ripening. Data are means of 35 fruits, *n* = 35. SE is given. Columns marked with one or two asterisks are significantly different from WT according to student’s t-test at *p*-value < 0.1 or 0.05, respectively

Transformed plants over-expressing *B’φ (b’φ*_*ox*_*)* showed no difference from original genotype (WT) regarding growth during the first weeks (Fig. 6a). Thereafter, differences became visible, and the *b’φ*_*ox*_ plants showed less vigorous growth compared with non-transformed plants (Fig. 6b, c). In 8-week-old *b’φ*_*ox*_ fresh weight of shoots and roots was reduced by 66 and 70%, respectively (Fig. 6d). Root length was similar in original genotype and *b’φ*_*ox*_, but number of leaves, stem height and stem thickness were reduced in *b’φ*_*ox*_. Although fruits had similar weight, the number of seeds was reduced by 60% per fruit (Fig. 6e).

**Fig. 7:**
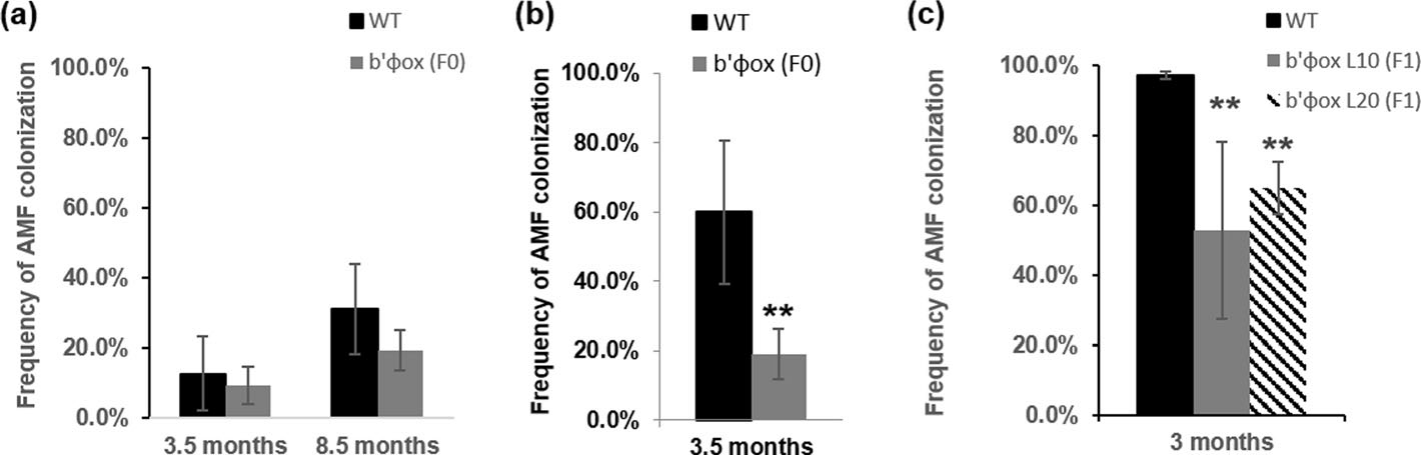
Frequency of root colonization in original genotype (WT) and *b’φ*_*ox*_ after inoculation with AMF. Colonization frequency for WT (black bars) and *b’φ*_*ox*_ (grey bars and hatched bar) (**a**) in soil 3.5 and 8.5 months after inoculation; (**b**) in vermiculite after 3.5 months; (**c**) in vermiculite after 3 months with another batch of *b’φ*_*o*x_ (F1 plants). Values are means ±SE from three plants. According to student’s t-test at *p*-value < 0.05, columns marked with two asterisks are significantly different from WT

To study mycorrhizal colonization, WT and *b’φ*_*ox*_ (F0, F1) were inoculated with AMF in soil and vermiculite. The frequency of AM in roots was assessed 3.5 months after adding AMF, and due to the low colonization, the assessment was repeated after 8.5 months for soil-grown plants (Fig. 7a). No significant differences in colonization frequency were found between WT and *b’φ*_*ox*_ in soil (Fig. 7a). The colonization frequency was higher in plants grown in vermiculite compared with those grown in soil, and the higher colonization frequency correlated positively with ABA levels, but negatively with *PP2A* expression, especially *B’φ* and *B’θ* (Figs. 1 and 5). A clear difference was seen between WT and *b’φ*_*ox*_ in three different experiments involving both *b’φ*_*ox*_ progenies F0 and F1. On average the colonization frequency was lowered by approximately 50% in *b’φ*_*ox*_.

### Gene expression in WT and *b’φ*_*ox*_

**Fig. 8:**
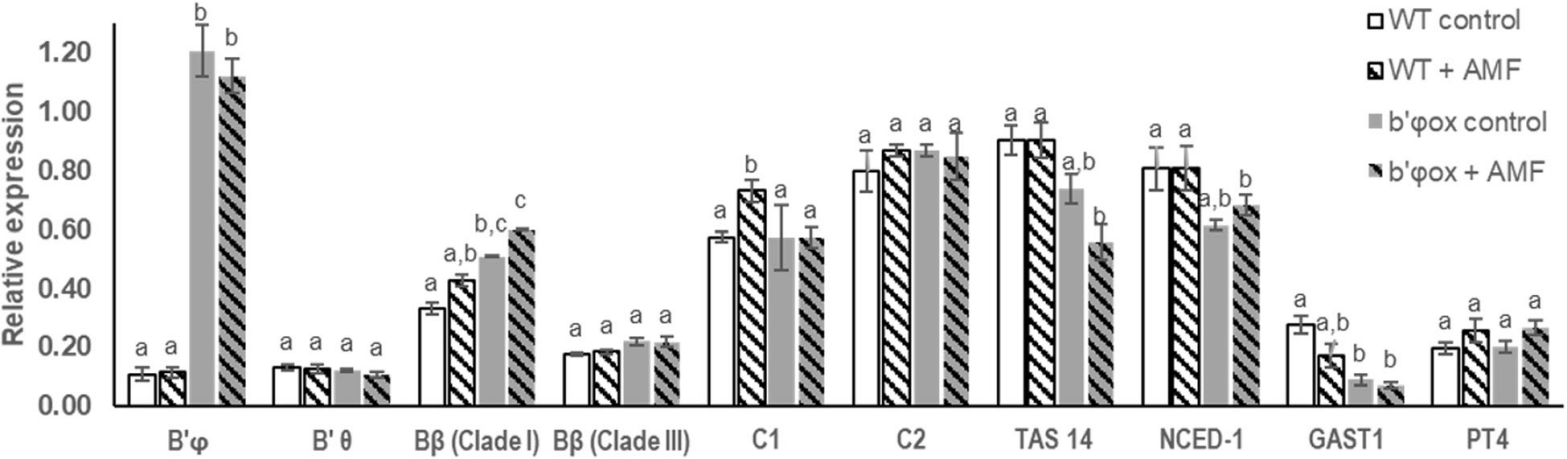
Expression analysis of PP2A subunits, hormone and AM-associated genes in roots of original genotype (WT) and *b’φ*_*ox*_ of non-treated and AMF-treated plants. WT (white, white-hatched columns) and *b’φ*_*ox*_ plants (grey, grey-hatched columns) had been grown for 3.5 months in vermiculite without (white or grey columns) or with AMF (hatched columns). The values are averages from three biological replicates normalized by the reference gene *ACTIN41*. For each gene, different letters represent significant different values according to one-way ANOVA and Tukey’s multiple range test, *n* = 3, (*p* < 0.05)

Expression levels of *B’φ* were, as expected, much higher in *b’φ*_*ox*_ than in WT, about 10-fold higher (Fig. 8). Over-expression of *B’φ* stimulated expression of the *Bβ* (clade I) gene in roots both treated (expression level up by 40%) and not treated with AMF (expression level up by 55%) (Fig. 8). For other PP2A subunit genes there were only small differences between WT and *b’φ*_*ox*_. Over-expression of *B’φ* led to decreased expression of both ABA reporter genes, *TAS14* and *NCED*, and the GA reporter gene *GAST1*. This strongly indicates that *B’φ* is important for regulation of the ABA/GA hormone balance in tomato roots. A lower ABA response in these plants is in agreement with less colonization by AMF.

## Discussion

### Hormone response reporter genes and PP2A

An ABA reporter gene *TAS14* showed almost no changes in expression when tomato roots were treated with three different PGPR strains (Fig. 2). On the other hand, reporter genes indicated that ABA levels were significantly decreased in roots of *B’φ* overexpressor plants treated with AMF compared with original tomato genotype (Fig. 8). The *b’φ*_*ox*_ plants showed lower expression also of a GA reporter gene. Clearly, the constitutive high expression of *B’φ* interfered with hormone actions in tomato roots.

#### RGPR and AMF regulate B′ expression

Bacteria or AMF treatment appeared to uphold the high expression of some PP2A genes, but decrease activity of other genes, and the most interesting results were found for the PP2A regulatory subunits *B’θ* and *B’φ*. The *B’θ* gene showed a striking response by being transiently down-regulated 2 and 24 h after bacterial treatment (Fig. 2). All three bacterial strains used, *P. simiae* (WCS417r), *A. brasilense* Sp245, and *A. brasilense* FAJ0009 triggered such a transient decrease in *B’θ* expression. The strongest effect was caused by *P. simiae*, and the effects were indifferent to auxin deficiency in the PGPR. Interestingly, plants that had been grown in soil with AMF also showed a low expression level of *B’θ* (Fig. 5a), indicating that down-regulation of *B’θ* is involved in plant-AMF interactions. However, it cannot be ruled out that adding AMF may also have stimulated growth of PGPR in the soil that in the next round would influence *B’θ* expression. The work indicated that *B’θ* was involved in the changes in morphology and in the frequency of root colonization associated with a complex crosstalk occurring between plants, AMF, and native soil bacteria. AMF inoculation inhibited *B’θ* expression only in soil-grown plants (where native bacteria were present), not in vermiculite-grown plants, and only soil-grown plants showed the canonical arbuscular form usually observed when roots are colonized by more than one AMF species [26]. The important role of *B’θ* is also supported by the observation that *B’θ* downregulation was related to the upregulation of the widely used AM colonization marker gene *PT4* (Fig. 5a). The lower basal expression level of *B’θ* in plants in vermiculite compared with soil may have contributed to the higher frequency of colonization (vesicular mycorrhizae) observed in vermiculite-grown plants. Previous work had pointed to the *Arabidopsis B’θ* and its two most closely related genes as being involved in biotic responses [8, 20]. The current results confirm that *B’θ* plays an important role in plant-microbe interactions, and here specifically point to an effect of plant growth-promoting microbes. In *Arabidopsis* plants with knocked out *B’θ*, proliferation of a pathogenic *Pseudomonas syringae* was decreased relative to WT plants [20]. A defence reaction against proliferation of pathogens implementing lowering expression of *B’θ* could possibly be a useful reaction for plant survival, and induced by PGPR and AMF.

### *B’φ* expression and phenotype of *B’φ* overexpressor plants

The most prominent results from the expression analysis of the *B’φ* gene was the very low expression level in all tissues and under all growth conditions investigated (Figs. 1, 2, 5, and 8, S1 Figure). Initially we attempted to make knock-out tomato plants using artificial microRNA to achieve gene silencing. However, in contrast to what had been found for the orthologue in *Medicago* spp*.* [17, 18], the amount of *B’φ* transcripts was very low in tomato. Therefore, selecting knock-out/down plants seemed unreasonable and technically difficult to verify further. We decided to make transformed plants over-expressing the *B’φ* gene to obtain information concerning functions of this gene. The over-expressor plants had a characteristic phenotype with smaller leaves, reduced stem thickness, reduced root and shoot fresh weight, and poor seed set per fruit (Fig. 6). Such changes are likely reflecting altered hormone levels, and both the ABA reporter genes and the GA reporter gene were down-regulated in *B’φ* over-expressor plants (Fig. 8). The observed phenotype could, at least partly, be explained by a low GA level [27, 28]. Formation of mycorrhizae had previously been linked to enhanced ABA levels [17]. Over-expression of *B’φ* appeared to inhibit AM formation (Fig. 7), and this could be related to the lowered ABA level in roots and disturbance of hormone balance as indicated by the hormone reporter genes (Fig. 8). The results obtained in the present work strengthen the view that *B’φ* has a role in regulation of AM formation. The present work also points to other functions of *B’φ* in growth and development. The Sol database [22] confirmed the very low expression levels for *B’φ in S. lycopersicum*; roots had non-detectable or very low expression levels for *B’φ*, about 500-fold lower than *C1* expression levels (Fig. S1a). In the Sol database, low levels of *B’φ* transcripts are also reported for *S. pimpinellifolium.* The interesting exceptions were tissues dissected with laser capture microdissection of ovary and fruit tissues combined with high-throughput RNA sequencing during early fruit development (0–4 days post anthesis) [29] (Fig. S1c). In ovules (day 0), *B’φ* expression was one third of *C1* and higher than for other *B′* subunits (*B’θ, B’κ, B’α, Bβ* -clade I) (Fig. S1c). This suggests that *B’φ* may have a function in the early fruit development, and hence also formation of seeds. Considering that *B’φ* has a function in early fruit development, the very high levels of ectopically expressed *B’φ* in the transformed plants (Fig. 8), may distort seed formation (Fig. 6). Taken together, the phenotype observations, our expression data and publicly available expression data support a function of *B’φ* in seed formation in addition to a role in mycorrhizae formation.

## Conclusions

The three applied bacteria strains strongly, and transiently, decreased expression of the PP2A regulatory subunit *B’θ*, pointing to a role for this gene in plant-PGPR interactions.

Work with *Arabidopsis* had previously indicated that plants with low *B’θ* expression were more resistant to pathogens than wild-type *Arabidopsis*. In further work with tomato, it would be interesting to design and test plants with altered *B’θ* expression to investigate if such changes could improve pathogen resistance in tomato. Analysis of the *B’φ* overexpressor plants and original cultivar substantiated a wider function of *B’φ* in growth and development in addition to a role in mycorrhization. High expression of the PP2A *B’φ* subunit gene decreased plant vigour, markedly reduced the number of seeds per fruit, and interfered with mycorrhization. In further work, PP2A interacting proteins (substrates) should be identified, and the involvement of PP2A in microbe recognition, versus establishing and upholding of symbiosis should be further explored. Expression of *B’φ* in more specific tissues, and studies on the role of *B’φ* at early stages of generative development should be performed.

## Methods

### Plant material

Seeds of *Solanum lycopersicum* cv. Heinz 1706-BG (LA4345) were obtained from the Tomato Genetic Resource Center (TGRC) at the University of California, Davis (http://tgrc.ucdavis.edu). Transgenic plants over-expressing *B’φ* were generated from hypocotyls of the Heinz cultivar using *Agrobacterium*-mediated transformation.

### Standard growing conditions for gene expression analysis in different plant organs

Tomato plants were grown in soil (75% potting soil and 25% vermiculite) in 0.5 L pots or in double autoclaved vermiculite in 0.4 L Magenta boxes (punctured at the bottom) at 22 °C in a 16 h light/8 h dark regimen. All plants were given Hoagland solution [30] at sowing containing macronutrients: 1 mM KH_2_PO_4_, 5 mM KNO_3_, 5 mM Ca (NO_3_)_2_, 2 mM MgSO_4_ and micronutrients: 2.6 mg/L H_3_BO_3_, 1.81 mg/L MnCl_2_x4H_2_O, 0.089 mg/L CuSO_4_x5H_2_O, 0.22 mg/L ZnSO_4_x7H_2_O, 0.029 mg/L H_2_MoO_4_x1H_2_O. Light was provided by fluorescent lamps (Osram L58W/77). Plants were watered weekly with tap water and (only for vermiculite) monthly with Hoagland solution. Root tissue, young leaves and flower buds of 5–12 mm were snap-frozen in liquid nitrogen and stored at − 80 °C. Samples were then homogenized in liquid nitrogen, and RNA was extracted.

### Plant growing conditions for PGPR experiment

Tomato plants were grown in 0.4 L Magenta boxes with (double) autoclaved vermiculite at 22 °C under artificial light in the 16 h light/8 h dark regimen and watered weekly with Hoagland solution. On day 41 after sowing, when plants had gained a sufficient root mass, equally developed plants were inoculated with 50 mL of bacteria suspended in 10 mM MgSO_4_, 5 × 10^7^ cells/mL for *Azospirillum* strains and 2.5 × 10^5^ cells/mL for *P. simiae* WCS417r. Control plants were given 10 mM MgSO_4_ only. Root tissue was harvested 2 h, 24 h, 1 week and 3 weeks after inoculation.

### Plant growth-promoting bacterial strains (PGPR), growth and inoculation

Three bacterial strains were used: *Azospirillum brasilense* Sp245 wild-type strain [31], its ipdC-knockout mutant FAJ0009 (Sp245 ipdC::Tn5) impaired in auxin biosynthesis [32, 33], and *Pseudomonas simiae* (formerly *Pseudomonas fluorescens*) WCS417r, a rifampicin-resistant strain derived from *Pseudomonas simiae* WCS417 originally isolated from the rhizosphere of wheat grown in Brazil [34]. For tomato inoculation, *P. simiae* WCS417r was cultured on King B medium [35] with 50 μg/mL rifampicin at 28 °C overnight. Colonies were loosened in 10 mL of 10 mM MgSO_4_ [34, 36], collected into a 15 mL Falcon tube and centrifuged at 4000 g for 5 min followed by 2 subsequent washes, the pellet was resuspended in fresh 10 mM MgSO_4_ to the appropriate concentration [37]. *Azospirillum* strains were cultured at 37 °C for 48 h on LB agar supplemented with 2.5 mM CaCl_2_ and 2.5 mM MgSO_4_. For FAJ0009, 50 μg/mL kanamycin was added. Colonies were used to produce an overnight culture in 5 mL of LB broth supplemented with 2.5 mM CaCl_2_, 2.5 mM MgSO_4_ at 37 °C shaking at 180 rpm overnight. The overnight culture (0.1 mL) was subcultured in 50 mL of appropriately supplemented LB broth and incubated under the same conditions. On the following day, the bacteria were pelleted and re-suspended in 10 mM MgSO_4_ to the concentration used for inoculation.

### Plant growing conditions for AMF experiments

The inoculum of AMF was obtained from the granular formulation under the commercial name “Rootgrow” (PlantWorks Ltd., Sittingbourne, UK) containing propagules of spores, hypha and root fragments colonized by *Funneliformis mossaeae*, *F. geosporus*, *Claroideoglomus claroideum, Glomus microagregatum, Phizophagus irregularis* [7]. Rootgrow, 1.5 mL granules, were added to the planting hole in each pot/Magenta box and covered with a thin layer of soil or vermiculite before sowing tomato seeds or planting seedlings. No granules (soil) or triple autoclaved granules (vermiculite) were added to the growing medium for control plants. The plants were grown at 22 °C in a 16 h light/8 h dark (soil) or 12 h light/12 dark (vermiculite) regimen and watered weekly with tap water (soil) or Hoagland solution (vermiculite) with ten times reduced phosphate concentration (0.1 mM PO_4_^3−^) for at least seven weeks or until plants showed profound signs of phosphate deficiency. Thereafter, the plants were watered with regular Hoagland weekly (soil and vermiculite). Two weeks prior to harvesting, the plants were watered only with tap water.

### Sample preparation for bright-field microscopy

Roots were washed with tap water, boiled in 10% KOH for 1 min and left in this solution overnight at room temperature. Roots were then rinsed with tap water and dipped in 3.7% solution of hydrochloric acid for 2–3 min. After removing the acid solution, staining solution was added. Staining solution was made from one volume of: 25% phenol, 25% lactic acid, 25% glycerol, 25% of 4 mg/mL trypan blue stock solution, plus two volumes of 95% ethanol [38]. Root tissue was placed in Eppendorf tubes, covered with the staining solution, and heated in a boiling water bath for 1 min with subsequent incubation on a shaker at room temperature for 4–16 h. After removing the staining solution, the roots were covered with a destaining solution (2.5 g/mL chloral hydrate) [39] and incubated for 6 h at room temperature before the solution was replaced with a fresh one and incubated overnight. The destaining solution was removed prior to covering the roots with 70% glycerol. Three slides with 20 stained root fragments, 5–8 mm, from each plant were examined under a light microscope with 10x and 100x magnification for AM structures such as spores, vesicles and arbuscules. The frequency of AMF colonization (F%) was calculated as a percentage of the root fragments with AM structures.

### Plasmid construct and agrobacterium preparation

To generate transgenic plants over-expressing *B’φ*, the full-length DNA sequence (1494 bp) of the *B’φ* gene (NCBI Reference Sequence: LOC101256045) was cloned into the pBA002 binary vector at the Xhol/SpeI sites [40] using flanking primers *B’φ*: forward primer (5′-TAGCACTCGAGATGACAAATTTTCTTGAT TCTGAGACAG-3′) and B’φ: reverse primer (5′-CCACTAGTTCACATTGCTG CATTTTCAATTTTTTCCC-3′). The pBA002 plasmid contains the cauliflower mosaic virus 35S promotor, which constitutively drives the expression of the transgene in all plant tissues at a high level [41]. The pBA002 plasmid also harbours spectinomycin resistance for selection in bacteria and the herbicide phosphinothricin (BASTA) resistance for selection in plants. To generate transgenic *b’φ*_*ox*_ plants, the pBA002-B’φ plasmid was transferred into the ABI-1 strain of *Agrobacterium tumefaciens* by the freeze-thaw procedure [42]. *Agrobacterium tumefaciens* ABI-1 strain, a derivative of the well-known GV3101 strain (pMP90RK) [43], was kindly provided by Dr. Amr Ramzy Abass Kataya, UiS, Norway. Prior to the tomato transformation, 5 mL of LB broth with 50 μg/mL kanamycin and 50 μg/mL spectinomycin was inoculated with *A. tumefaciens* and incubated at 28 °C on a shaker at 200 rpm for two days, then 1 mL was subcultured in 100 mL of LB broth containing the same antibiotics and incubated for about 24 h under the same conditions until OD_600_ reached 1. The bacteria were pelleted and resuspended in liquid MS medium [44] to OD_600_ = 0.2 [45] and used for the tomato transformation.

### Plant tissue and transformation by *Agrobacterium*

Tomato seeds were surface sterilized with 75% ethanol for 1 min and 15% hydrogen peroxide for 15 min, rinsed with water, germinated on MS medium (4.3 g/L MS salts (Sigma-Aldrich, USA), 3% sucrose, 0.8% agar, pH 5.8) and cultivated at 22 °C in a 16 h light/8 h dark regimen for 20 days or until the cotyledons had opened completely. Three days before transformation the hypocotyls were cut into 7–10 mm explants and placed on pre-culture medium (MS salts, 3% sucrose, vitamins, 0.5 mg/L indole-3-acetic acid (IAA), 1 mg/L benzylaminopurine (BAP), 0.7% agar, pH 5.8) [46] and incubated for 72 h in the dark at 27 °C [44, 45]. For transformation, all the explants were immersed in the *Agrobacterium* suspension and shaken for 20 min at room temperature, blotted dry and transferred to the co-cultivation medium (MS salts, vitamins, 3% sucrose, 0.7% agar, 0.5 mg/L IAA and 1 mg/L BAP) and incubated for two days in the dark at room temperature [44, 46]. Explants were then placed on shoot induction medium (MS salts, vitamins, 3% sucrose, 0.7% agar, 250 mg/L cefotaxime, 250 mg/L carbenicillin, 10 mg/L BASTA, 0.5 mg/L IAA and BAP 2 mg/L) for further cultivation at 22 °C with 16 h photoperiod for 90 days, subcultured to fresh medium every 30 days. Explants developing Basta-resistant calli produced shoots. The shoots were excised from the calli and transferred to root induction medium (MS salts, vitamins, 3% sucrose, 0.7% agar, 250 mg/L cefotaxime, 250 mg/L carbenicillin, 0.5 mg/L IAA, 10 mg/L BASTA) and cultivated for 30 days. Plants developed from the tomato explants were genotyped using Phire® Plant Direct PCR Kit (Thermo Fisher Scientific, Waltham, USA) and transferred either to vermiculite or soil for further growth. The transformed plants obtained from the explants were considered as F_0_ progeny of *b’φ*_ox_. Transgenic lines from F_0_ and F_1_ progenies were used for further analyses.

### Semi-quantitative RT-PCR analysis

Total RNA was isolated from roots, leaves or flower buds using RNeasy® Plant Mini Kit (Qiagen, Hilden, Germany), and cDNA was made using SuperScript™ VILO™ cDNA Synthesis Kit (Invitrogen by Thermo Fisher Scientific, USA). Polymerase chain reaction (PCR) was performed in 10 μL reactions using DreamTaq DNA Polymerase kit (Thermo Fisher Scientific, Vilnius, Lithuania). The PCR products were separated on agarose gel and quantified using Bio-Rad Image-Lab 6.0 Software. The average band intensities from three plants was used to calculate a transcript level. Relative quantification of the transcript levels was based on normalization of the target gene band densities with respect to the reference gene *ACTIN41*. The primer sequences for semi-quantitative RT-PCR are listed in Supplementary Table 1.

### Statistical analysis

Data were analysed by student’s t-test using the Excel statistical package (version Microsoft 385) or one-way ANOVA with Tukey’s multiple range test using the IBM SPSS Statistics 26.

## Supplementary Information

**Additional file1: Table S1.** List of primers. **S1 Fig.** Expression of PP2A subunit genes in *S. lycopersicum* roots and leaves, and *S. pimpinellifolium* ovules and leaves. **S2 Fig.** Visual phenotype of tomato plants three weeks after treatment with PGPR.

## Data Availability

All data used are available in the article and supporting information.
