# Supplementary material for: Pinpointing regulatory protein phosphatase 2A subunits involved in beneficial symbiosis between plants and microbes
Source: BMC Plant Biol. 2021 Apr 16;21:183. doi: 10.1186/s12870-021-02960-4 (PMC8052836; doi:10.1186/s12870-021-02960-4)
Supplement: Supplementary file 1 — Additional file1: Table S1. List of primers. S1 Fig. Expression of PP2A subunit genes in S. lycopersicum roots and leaves, and S. pimpinellifolium ovules and leaves. S2 Fig. Visual phenotype of tomato plants three weeks after treatment with PGPR. [file 12870_2021_2960_MOESM1_ESM.pdf]

Supporting information

Pinpointing regulatory protein phosphatase 2A subunits involved in beneficial symbiosis between plants and microbes

Irina O. Averkina[1], Muhammad Harris[1,2], Edward Ohene Asare[1], Berenice Hourdin[1], Ivan A. Paponov[3], Cathrine Lillo[1*]

1 IKBM, Department of Chemistry, Bioscience and Environmental Engineering University of Stavanger, 4036 Stavanger, Norway

2 Faculty of Veterinary Medicine, Norwegian University of Life Sciences, 0454 Oslo, Norway

3 NIBIO, Norwegian Institute of Bioeconomy Research, Division of Food Production and Society, P.O. Box 115, NO-1431 Ås, Norway

Current address: Department of Food Science, 8200 Aarhus University, Aarhus, Denmark

*Corresponding author

Table 1. List of primers

Target gene	Sequence (5'- 3')	Annealing (°C)	PCR Product Size (bp)
pBA002 cloning vector [a,b]<br><br>pBA002-B'φ ox insert [a,b]	Forward:<br>CGTCTTCAAAGCAAGTGGATTGATG<br>Reverse:<br>TGCTTAACGTAATTCAACAACAGAAATTAT	54	276<br><br>1762
B'φ<br>PP2A-B'φ regulatory subunit (M. A. Booker & A. DeLong, 2017) [a]<br>NCBI Reference Sequence: XM_010317091.2	Forward:<br>ATGACAAATTTTCTTGATTCTGAGACAG ATCG<br>Reverse:<br>TCACATTGCTGCATTTTCAATTTTTTCCC	54	1486
B'φ<br>PP2A-B'φ regulatory subunit [b]<br>NCBI Reference Sequence: XM_010317091.2	Forward:<br>GAGACTGATCAAAGGCACCCTGGAATCG<br>Reverse:<br>ACAATGCGCGTTCAGCAACCTGCGAG	61	406
B'θ<br>PP2A B56 (Clade 6/9/10) [b]<br>NCBI Reference Sequence: XM_004239128.4	Forward:<br>AGGTGTATGGTGCCGTTGTT<br>Reverse:<br>GATGGCACAAGCACAGCTTCCA	55	389
Bβ (Clade I)<br>PP2A B55 (CLADE I, Booker 2017) [b]<br>NCBI Reference Sequence: XM_004241456.4	Forward:<br>CTCACATGGAGTCTTCCCCG<br>Reverse:<br>AAGCCACATGCAGCAACTT	55	373
Bβ (Clade III)<br>PP2A B55 (CLADE III) (M. A. Booker & A. DeLong, 2017) [b]<br>NCBI Reference Sequence: XM_004252324.4, XM_019211570.2, XM_010316006.3<br>(transcript variant X1, X2, X3)	Forward:<br>GTGGTGATGGTTCGCGAGTA<br>Reverse:<br>CATTTGCATCAACACCGGCA	56	213
B' κ<br>PP2A-B56 (Clade B11) [b]<br>NCBI Reference Sequence: XM_004251542.4 (transcript variant X1), XM_010315410.3 (transcript variant X2), XM_010315411.3 (transcript variant X3), XM_026028819.1 (transcript variant X4), XM_026028820.1 (transcript variant X5)	Forward:<br>ACCTCTGCACAAGCCAAAGT<br>Reverse:<br>TCCAGTGGTTCTGGCTGTTC	56	392
A βI<br>B65 or PP2A-A [b]<br>NCBI Reference Sequence: XM_004236914.4 (transcript variant X1), XM_010321123.3 (transcript variant X2)	Forward:<br>TAGCAAAGGACAGAGTGCCC<br>Reverse:<br>ACTGTTTCCTGGTGTGACGG	56	291

A βII<br>B65 or PP2A-A [b]<br>NCBI Reference Sequence:<br>XM_004238897.4 (transcript variant X1),<br>XM_019214032.2 (transcript variant X2)	Forward:<br>TGCACCAGTAATGGGCTCTG<br>Reverse:<br>ACTGGGCCGAATGGTTTCT	56	166
**Target gene**	**Sequence (5’- 3’)**	**Annealing (°C)**	**PCR Product Size (bp)**
C1<br>PP2A catalytic subunit (PP2Ac1) [b]<br>NCBI Reference Sequence:<br>NM_001247587.2	Forward:<br>GCGGCAACGTCCTGATTACTA<br>Reverse:<br>AGGCTCAATCTGTCGTGGAG	56	667
C2<br>PP2A catalytic subunit (PP2Ac2) [b]<br>NCBI Reference Sequence:<br>NM_001246875.2	Forward:<br>ATGCCGTCTCATGCAGATCTA<br>Reverse:<br>CTTCCTTGTGGTGTCGGGCTCTA	54	900
TAS 14<br>Abscisic acid and environmental stress-inducible protein [b]<br>NCBI Reference Sequence:<br>NM_001247109.1	Forward:<br>CATCACCATGAGGGGCAACA<br>Reverse:<br>GCGTCAGCACACTTTACACG	56	363
NCED-1<br>Nine-cis-epoxycarotenoid dioxygenase 1 [b]<br>NCBI Reference Sequence:<br>NM_001247526.2	Forward:<br>GTGCAGAAAGCAGCAGCAAT<br>Reverse:<br>CGTAAACCACCGGTGAACCT	55	871
GA3ox-1<br>Gibberellin-3β-hydroxylase-1 [b]<br>NCBI Reference Sequence:<br>AB010991.1	Forward:<br>AGGCCCAAAAGGTGAAACCA<br>Reverse:<br>CCGTACGGATGAAAGTGCCT	56	463
GA3ox-2<br>Gibberellin-3β-hydroxylase-2 [b]<br>NCBI Reference Sequence:<br>NM_001246926.3	Forward:<br>AGGCCCAAAAGGTGAAACCA<br>Reverse:<br>CCGTACGGATGAAAGTGCCT	51	276
GA20ox-1<br>Gibberellin 20-oxidase-1 [b]<br>NCBI Reference Sequence:<br>NM_001247141.1	Forward:<br>AGGCCCAAAAGGTGAAACCA<br>Reverse:<br>CCGTACGGATGAAAGTGCCT	51	451
GA20ox-2<br>Gibberellin 20-oxidase-2 [b]<br>NCBI Reference Sequence:<br>NM_001247699.2	Forward:<br>CTACAAGGCTCGTCCCACAT<br>Reverse:<br>TGAAAGCGCCATAAATGTGTC	51	402
GA20ox-3<br>Gibberellin 20-oxidase-3 [b]<br>NCBI Reference Sequence:<br>NM_001247650.2	Forward:<br>CCGGACCATGAGAAGCCAAA<br>Reverse:<br>GATAGTGCCATAAACGTGTCGC	55	752
GA20ox-4<br>Gibberellin 20-oxidase-4 [b]	Forward:<br>CTACAAGGCTCGTCCCACAT		

NCBI Reference Sequence: NM_001247434.2	Reverse: TGAAAGCGCCATAAATGTGTC	53	375
GAST1 Gibberellin responsive gene NCBI Reference Sequence: NM_001309377.1	Forward: TCAGGTTTCAAGGGCCAACA Reverse: CACTTTGGGCCACCTCTTTTG	56	275
Actin 41 Reference gene [b] NCBI Reference Sequence: NM_001330119.1	Forward: CACTGTATGCCAGTGGTCGT Reverse: GCATCTCTGGTCCAGTAGGAAA	58	773

[a]Primers used with **Phire® Hot Start II DNA Polymerase**
[b] Primers used in sqRT-PCR analysis

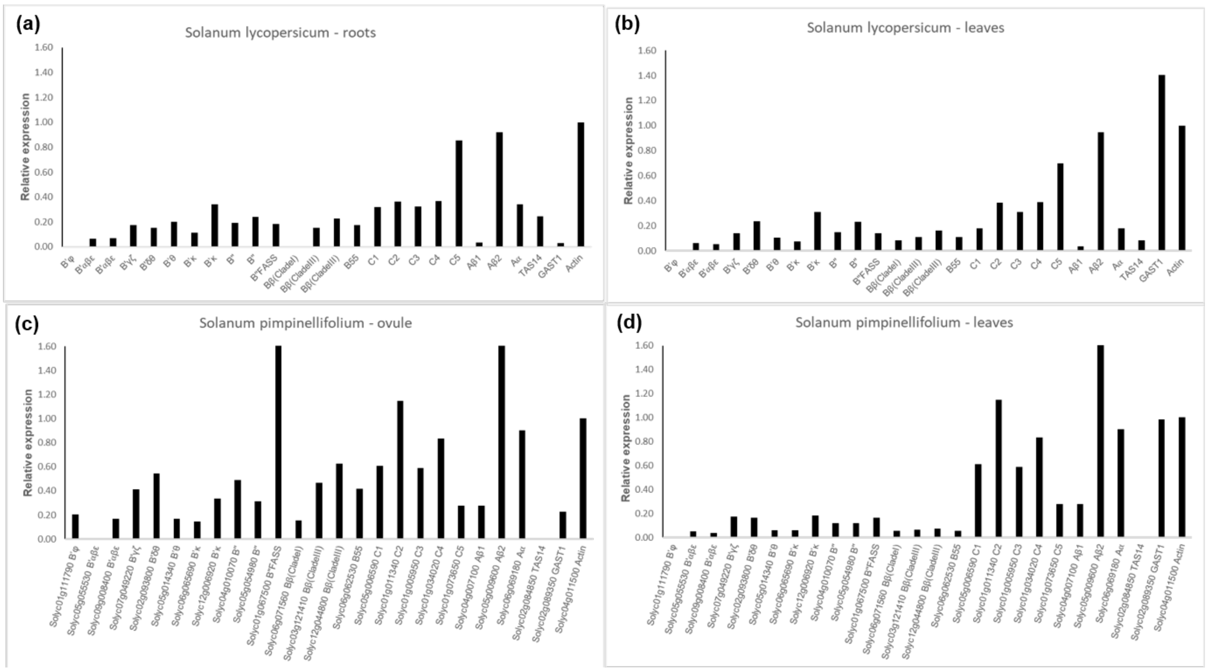

S1 Figure. Expression of PP2A subunit genes in *S. lycopersicum* roots and leaves, and *S. pimpinellifolium* ovules and leaves. Publicly available data from the Sol genetics database. Genes presented are named according to International Tomato Annotation Group, and orthologues in Arabidopsis. The genes presented are PP2A subunits: eight different B', three B'', four B/B55, five C and three A (Aβ2 is 2.23, exceeding the scale), additionally TAS14, GAST1 and Actin. All are normalized to Actin 41 (set to 1).

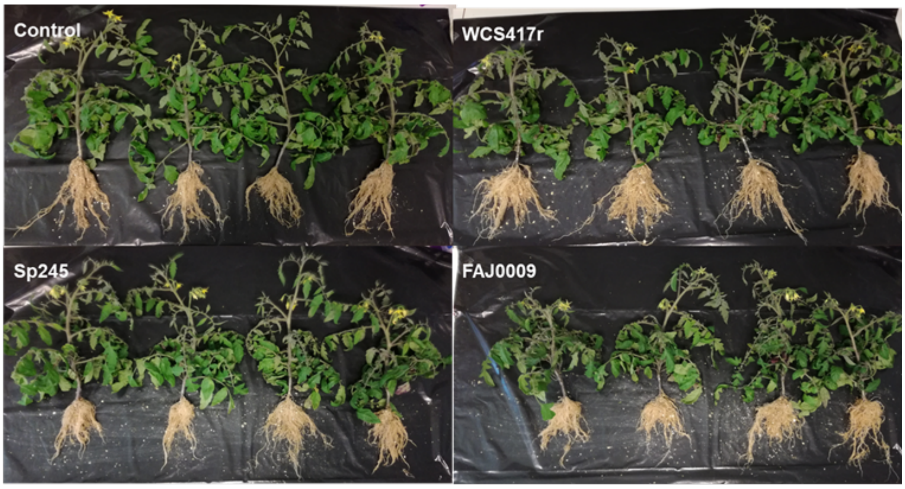

S2 Figure. Visual phenotype of tomato plants three weeks after treatment with PGPR.
Two-month-old tomato plants not cultivated (upper left photo) and cultivated with WCS417r (upper right photo); Sp245 (lower left photo) and FAJ0009 (lower right photo).
